# Discovery of a Digenean (Cryptogonimidae) Living in a Cleft-Lipped Goby, *Sicyopterus cynocephalus* (Teleostei: Gobiidae) from Ranongga Island, Solomon Islands: Analysis of Multiple Ribosomal DNA Regions

**DOI:** 10.3390/pathogens12070923

**Published:** 2023-07-09

**Authors:** Patrick D. Mathews, Nicolas Rabet, Luis L. Espinoza, Vincent Haÿ, Céline Bonillo, Philippe Keith, Clara Lord, Fabienne Audebert

**Affiliations:** 1 Unité Biologie des Organismes et Écosystèmes Aquatiques-BOREA, Sorbonne Université, Muséum National d’Histoire Naturelle, CNRS, IRD, UCN, UA, CP 26, 43 Rue Cuvier, 75005 Paris, France; nicolas.rabet@sorbonne-universite.fr (N.R.); hvincent75@gmail.com (V.H.); celine.bonillo@mnhn.fr (C.B.); philippe.keith@mnhn.fr (P.K.); clara.lord-daunay@sorbonne-universite.fr (C.L.); 2 Laboratory of Biology and Molecular Genetics, Faculty of Veterinary Medicine, National University of San Marcos, Lima 2800, Peru; llunae@unmsm.edu.pe

**Keywords:** digenea, Gobiidae, *Sicyopterus cynocephalus*, freshwater, Solomon Islands

## Abstract

This study results from a continued investigation of the occurrence and diversity of parasites of freshwater fish in the Solomon Islands. Thus, we revealed a new host as well as a new site of infection and a new geographical area for the cryptogonimid parasite, *Stemmatostoma cribbi* (Digenea: Cryptogonimidae). The cryptogonimid species was identified based on general morphology and on molecular data of metacercariae found in the gills of the cleft-lipped goby, *Sicyopterus cynocephalus,* from Ranongga Island, Western Province of the Solomon Islands. This is the first report of a *Stemmatostoma* sp. digenean parasitizing fish of the genus *Sicyopterus* in the Indo-Pacific region and the first report of *S. cribbi* infection in a fish from the Solomon Islands. Phylogenetic analysis performed by Bayesian inference and maximum likelihood confirmed the presence of the cryptogonimid in a well-supported subclade of *Stemmatostoma* spp.

## 1. Introduction

Tropical island streams of the Indo-Pacific are colonized by diadromous species which alternate between freshwater and seawater environments. There are three different types of diadromy, such as catadromy for organisms that mature in freshwater and reproduce at sea (e.g., many eel species), anadromy for organisms that mature at sea and reproduce in freshwater (e.g., salmon), and amphidromy in which freshwater to sea migration is not linked to reproduction. Indeed, amphidromous species live, grow and reproduce in freshwater; the eggs hatch in a river, and larvae have a few hours to migrate downstream to the sea, where they spend 1 to 6 months, depending on the species. After this marine phase, post-larvae return to the rivers, where they migrate upstream to colonize the adult habitat. Returning larvae are targeted in many countries by local fishing activities, and Sicydiinae post-larvae represent a high-value consumption fish largely marketed in the Indonesian food market [1]. 

Most of the species that live in tropical island streams have an amphidromous life cycle, whether they are mollusks, decapod crustaceans, or teleosteans, like the Sicydiinae gobies [2]. The amphidromous life cycle is an adaptation to the colonization of tropical islands, which are distant from one another and from continental landmasses. In addition, such island streams are subject to extreme climatic and hydrological seasonal variations [2]. The amphidromous life cycle ensures a pool of larvae at sea and helps maintain tropical island stream biodiversity. 

Sicydiinae Bleeker, 1874 represents most of the fish diversity in insular systems, where they comprise about 100 described species [3] and have the highest levels of endemism [2,3,4,5]. Among these amphidromous Sicydiinae gobies, *Sicyopterus* Gill, 1860 is one the most diverse genera, with 24 species described from the Indo-Pacific tropical islands [2,6]. Among the known *Sicyopterus* species, *Sicyopterus cynocephalus* Valenciennes, 1837 is found in the Western Pacific, Papua New Guinea, Indonesia, the Australian wet tropics, and the Solomon Islands [2,7]. The Western Province of the Solomon Islands is made up of 35 islands, which are part of the New Georgian chain of islands. This region has a wide range of habitats ranging from high-elevation mountain forests to low-lying atolls and coral reef systems [8]. This region represents a hotspot for conservation and is identified with high alpha diversity of fish and marine vertebrates [8]. Approximately 80 freshwater fish species inhabit Solomon Island streams [9]. Studies on freshwater biodiversity in the Solomon Islands are relatively recent, and research on the parasite taxa is only just starting. Although a new species of cnidarian parasite found on an eel host (*Anguilla marmorata*) from the Solomon Islands was recently described [10], there remains a vast gap in the knowledge of the species richness of parasite taxa and their interactions with the freshwater fishes that inhabit this diverse tropical Indo-Pacific bioregion [10]. 

In Indo-Pacific freshwater environments, few surveys of the parasite fauna of freshwater fish have been reported. In a recent study in the Australian Wet Tropics, adult Cryptogonimids (Cryptogonimidae Ward, 1917), *Stemmatostoma cribbi*, were found in Terapontidae and Kuhliidae definitive hosts [11]. Both of these latter fish families are found in the Solomon Islands, and species like *Kuhlia rupestris* (Kuhliidae), also called the jungle perch, are fished by local populations for consumption (pers. obs). Both jungle perch and the Sicydiinae cleft-lipped goby *S. cynocephalus* are common in Solomon Islands' freshwater systems.

With 66 recognized genera, the Cryptogonimidae comprise a large family of digenean trematodes distributed worldwide [12,13,14]. Cryptogonimids parasitize marine and freshwater fishes [15,16,17,18,19], as well as reptiles in many regions around the world [12,15]. In their complex life cycle, the adult trematode is typically found in the fish intestine and passes embryonated eggs, which must be eaten by the first intermediate host, a snail. Within the snail host, sporocyst generation occurs, which is followed by redial generation and, in turn, by cercarial generation. The cercariae escape the snail and penetrate a fish as a second intermediate host in which they encyst as metacercariae. Metacercariae may survive up to 6 months and will develop into the adult form after the second intermediate host fish is consumed by a definitive host, such as Kuhliidae or Terapontidae, or even a reptile [18]. Although an impressive number of cryptogonimid trematodes are known to infect many fish species across different continents [14,15,20], cryptogonimid fauna and other digenean families have been poorly explored in the Solomon Islands. 

A field mission in the Solomon Islands was undertaken in November 2016 to inventory fish and crustaceans in the Western Province. During this field mission, as an exploratory study, we sampled one of the most common species of goby, *S. cynocephalus,* to investigate and catalog the parasite fauna of this species. Indeed, parasites of Sicydiinae have never been studied, although this family is well represented in Indo-Pacific stream ecosystems and is an important source of food in many regions.

This study aimed to increase knowledge on the occurrence and characterization of parasites of fish from the Solomon Islands and to initiate a study on parasites in tropical island streams of the Indo-Pacific. In this first study, we show for the first time that *S. cynocephalus* is an intermediate host in the life cycle of *S. cribbi* and report new data that expands the host range, sites of infection, and geographical distribution of *S. cribbi*. In addition, we provide details of the remarkable genetic uniformity of this parasite through comparative genetic analyses.

## 2. Materials and Methods

### 2.1. Host and Parasite Collection

Twenty-five wild specimens of *S. cynocephalus* were obtained during the field mission in the Western Province of the Solomon Islands in 2016. The fish were caught using an electrofishing system DEKA 3000 (Geratebäu, Marsberg, Germany) in the Poro River (8°03.082′ S, 156°35.711′ E) on Ranongga Island and euthanized using an overdose of clove essential oil (10%). After euthanasia, fish were weighed with a professional digital mini-scale Brifit (Dusseldorf, Germany). Total length was measured to the nearest tenth of a millimeter with a Powerfix dial caliper (Bedfordshire, UK). The gills and digestive tract of each fish were removed and preserved in 95% ethanol, and the rest of the fish was also preserved in 95% ethanol. Specimens were then transported to the laboratory of Ichthyology of the Museum National d’Histoire Naturelle, Paris, France, for complete parasitological examination.

The external body of each fish, the entire digestive tract, internal cavity, muscles, and all gills were inspected using a binocular Nikon PN-TSE 30 (Japan). Cryptogonimid cysts were found on the gills and taken for morphological analysis. Because the primary objective of the field mission was not aimed at studying fish parasites, 95% ethanol was the only fixative available to preserve the specimens. Therefore, live parasites could not be fixed according to the protocol described by Cribb and Bray (2010). For morphological analysis, whole cryptogonimid cysts were processed according to standard methods [21,22] and examined on slide mounts under a stereo microscope Olympus SZX7 with an SC50 Olympus camera, driven by Stream software, and under an Olympus BX51 microscope with an MC170 Leica camera, driven by LAS software (Rungis, France). The morphological identification of cryptogonimid trematodes was performed by comparing them to *S. cribbi* adult worms described by Miller and Adlard [11] and to *S. pearsoni* metacercaria described by Cribb [21]. Drawings of encysted metacercaria were made using the pictures from the same microscope and digitized using a drawing pad with Adobe Illustrator CC 2018. Measurements in micrometers of encysted metacercaria were made using an ocular micrometer.

Five encysted metacercaria were found, photographed, measured, and used for molecular analysis. Unfortunately, given the small amount of material, it was not possible to put the specimens in a collection.

### 2.2. Molecular Analysis

For precise host identification, DNA was extracted from pectoral fin tissue using a DNeasy^®^ Blood & Tissue Kit (Qiagen, Valencia, CA, USA) in accordance with the manufacturer’s instructions. The mitochondrial DNA gene *cytochrome c oxidase subunit I* (*COI*) was amplified by PCR using primers (FishF1 TCAACCAACCACAAAGACATTGGCAC and FishR1 TAGACTTCTGGGTGGCCAAAGAATCA, Ward et al. [23]) as described in Mathews et al. [10]. *COI* Sanger sequencing was performed in both directions by a commercial vendor (Genoscreen, Lille, France). 

For molecular analysis of encysted metacercaria, all the whole specimens stored in 95% ethanol were processed for DNA extraction using the QIAamp DNA Micro kit (Qiagen, CA, USA) in accordance with the manufacturer’s instructions. The library preps from DNA were performed with a Nextera XT kit (Illumina^®^) (San Diego, CA, USA): fragmentation and Illumina adapter and index ligation. Equimolar pools of each library were established. Qualification and quantification of the final library were established before sequencing on an Illumina Miseq^®^ instrument (San Diego, CA, USA) with 25 million reads per run of 2 × 300 bases each (32 libraries per run). The 18S rRNA coding region was initially found by local BLAST, and raw sequences were progressively assembled and annotated using Geneious^®^ 11.1.4. Some partial mitochondrial genes were similarly isolated using NC_049068 (*Diplostonum ardeae*). This method allowed reconstitution of a mitochondrial genomic fragment of 7431 bp that included External Transcribed Spacer (ETS) (698 bp), 18S (1942 bp), Internal Transcribed Spacer 1 (ITS1) (554 bp), 5.8S rDNA (157 bp), ITS2 (299 bp) and 28S rDNA (3781 bp) (accession number: OQ968484). A fragment of mitochondrial genome was also obtained that included part of the 16S rRNA coding region (213 bp) (accession number: OQ974706), a partial *cytochrome c oxidase I* (*COI*) sequence (322 bp) (accession number: OQ969991) and a partial *cytochrome B* (*CytB*) sequence (484 bp) (accession number: OQ968485). A basic local nucleotide alignment search (BLASTn) was conducted to verify the similarity of the sequences we obtained with other cryptogonimid sequences available in the GenBank database [24]. To visually assemble sequences and to compare the sequences obtained with the most closely related cryptogonimid species, as determined by the BLAST search, the biological sequence alignment editor Bioedit was used [25]. 

Phylogenetic analysis of the partial 28S rDNA region of the cryptogonimid species sequenced here was performed using maximum likelihood (ML) and Bayesian inference (BI). ML was conducted with a Kimura 2-parameter (K2P) evolution sequence model in the MEGA 6.0 program [26]. Bootstrap analysis (1000 replicates) was employed to assess the relative robustness of the tree branches. *Galactosomum lacteum* (Accession number AY222227), *Galactosomum bearupi* (Accession number MH257773), and *Euryhelmis costaricensis* (Accession number AB521800) sequences were used as an outgroup. BI analyses were conducted with MrBayes software version 3.2 [27], as described by Miller and Adlard [11]. BI analyses were run over 10,000,000 generations (ngen = 10,000,000) with 2 runs, each containing 4 simultaneous Markov Chain Monte Carlo chains (nchains = 4) and every 1000th tree saved (samplefreq = 1000). The model was set as “lset nst = mixed” and “rates = invgamma”, allowing sampling across substitution models and in a proportion of invariable sites. The pairwise method with the p-distance model in MEGA 6.0 [26] was performed on 28S rDNA, 5.8S rDNA, and ITS-2 to evaluate the genetic divergence among the cryptogonimid species that clustered together with the new *S. cribbi* sequences. 

## 3. Results

**Figure 1:**
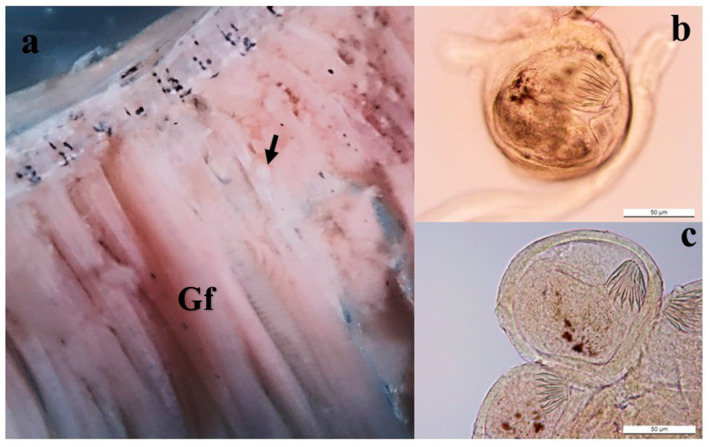
Gill infected with trematode cyst. (**a**) Gill filament (Gf) showing macroscopically visible cyst (black arrow). (**b**,**c**) Encysted metacercaria in frontal view with body folded on itself, showing 14 circumoral spines. Scale bars: 50 µm.

Out of 25 wild specimens of *S. cynocephalus* examined (mean total length: 59.8 ± 10.6 mm; mean weight: 3.67 ± 1.49 g), cysts were found in only one specimen. These cysts were found in the gill filaments only (Figure 1a) and not in any other organs, muscles, or tegument. A total of 5 cysts, enclosed by a sheath of host gill tissue, were found. Each cyst contained one metacercaria (Figure 1b,c).

The mean diameter (range) of the whole cysts was 135.4 µm (113–179 µm). Length, width, circumoral spine length, and width of oral and ventral suckers of the encysted metacercaria are given in Table 1.

**Figure 2:**
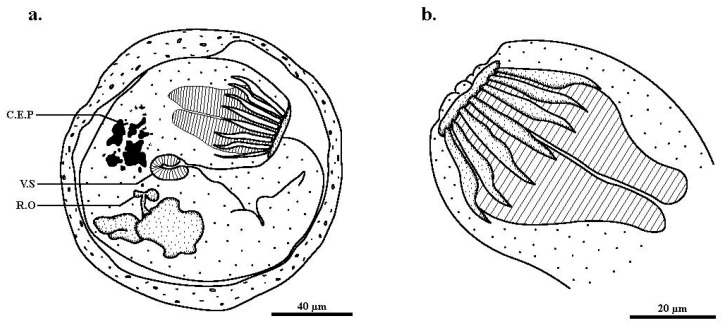
Metacercaria of *Stemmatostoma cribbi* found in *Sicyopterus cynocephalus* gills. (**a**) Encysted metacercaria. (**b**) Excysted oral sucker with 7 out of 14 circumoral spines visible. C.E.P.: cercaria eyespot pigment; R.O.: Rudimentary ovary; V.S.: ventral sucker.

Within the cyst, the metacercarian body was folded on itself (Figure 2a). The excysted individuals presented an oral and a ventral sucker, which were retracted, cercaria eyespot pigments, and a rudimentary ovary indicative of a larval stage. The presence of fully developed circumoral spines indicates a metacercaria stage (Figure 2b). The 14 oral spines identified these specimens as *Stemmatostoma cribbi* since this species is distinguished morphologically from the only other species in the genus, *Stemmatostoma pearsoni,* by having consistently fewer oral spines (14 for *S. cribbi* vs. 16 for *S. pearsoni*).

**Figure 3:**
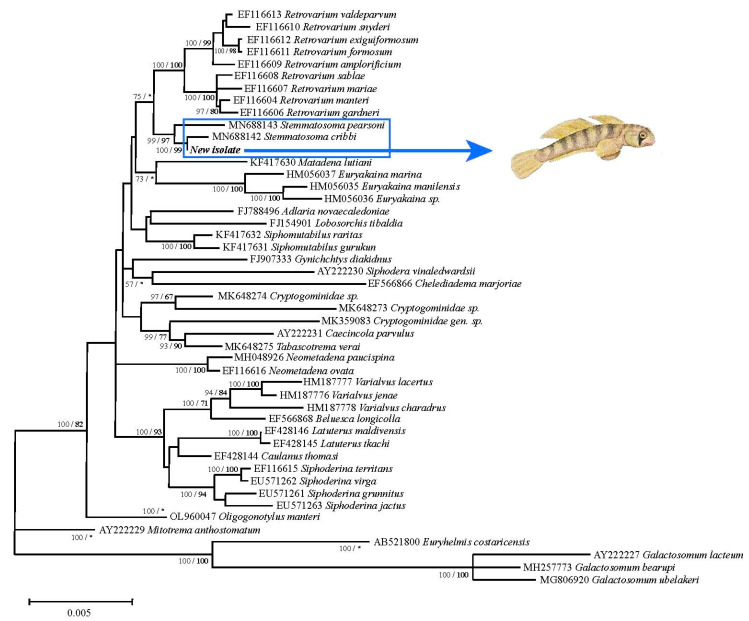
Phylogenetic tree based on Bayesian inference and Maximum Likelihood analyses of the partial 28S rDNA gene sequence. Nodes are supported by 1000 replicates of bootstrap from Maximum Likelihood and by posterior probability from Bayesian Inference. GenBank accession numbers are given before the name of the species.

Based on molecular data, the BLAST search revealed a high similarity between the newly-obtained 28S rDNA gene sequence and a previously-published sequence of *S. cribbi* (query cover 100%, maximum identity 99.69%), a known parasite of the terapontid fishes *Hephaestus fuliginosus* and *Hephaestus tulliensis*, and the kuhliid fish *Kuhlia rupestris* from Queensland, Australia. The pairwise comparison between the new isolate from the Poro River in the Solomon Islands and a previously deposited 28S rDNA gene sequence of *S. cribbi* found an overall genetic divergence of 0.6% over the 1253 bp, with just eight nucleotide base changes between the two sequences. Similarly, sequencing of the partial 5.8S and entire ITS-2 rDNA regions of the newly-isolated specimens and *S. cribbi* sequences revealed only five nucleotide differences between both sequences. The phylogenetic trees generated by ML and BI showed congruent topologies, and the new sequences obtained appeared in a well-supported subclade (Bootstrap ML 100/99 BI) with the previously available *S. cribbi* sequence, while the two latter, along with *S. pearsoni* Cribbi, 1986 also form a well-supported subclade (Bootstrap ML 99/97 BI) (Figure 3). 

## 4. Discussion

Despite considerable knowledge of fish biodiversity in the freshwater ecosystems of the Solomon Islands (about 80 fish species) [4], little is known about the species richness of parasite taxa infecting the freshwater fish of this diverse bioregion. Indeed, only one parasite species has been described in the giant mottled eel (*Anguilla marmorata*) [10]. To our knowledge, this is the first report of a *Stemmatostoma* species digenean trematode parasitizing fish of the genus *Sicyopterus* in the Indo-Pacific region and the first report of an *S. cribbi* infection in a fish from the Solomon Islands. These findings add to our knowledge about the host-parasite interactions of this cryptogonimid trematode, as well as the fish parasite diversity in the Solomon Islands archipelago. 

Prior to this study, adult *S. cribbi* had been reported to infect the intestine and pyloric caeca of freshwater fish of the families Terapontidae and Kuhliidae from Australia [11]. Terapontidae and Kuhliidae are families that are commonly present in the Solomon Islands [21]. Cribb [21] reported that some Gobioids are second intermediate hosts of *S. pearsoni*, a species closely related to *S. cribbi*, with metacercaria present. Our finding of *S. cribbi* metacercaria in *S. cynocephalus* gills demonstrates that this Sicydiinae goby is a second intermediate host for *S. cribbi.* Furthermore, the infected gill tissue observed in our study represents a new site of infection for this cryptogonimid parasite. Indeed, metacercarian cysts of *S. pearsoni* are usually found in the general body musculature, the roof and floor of the mouth, the opercula, and the fins, but not in the gills. 

Regarding the transmission of *S. cribbi* in a wild specimen of *S. cynocephalus*, direct contact with infective cercariae that emerged from small freshwater snails is very likely the source of infection evidenced in this study. The presence of these metacercariae encysted on the gills externally is consistent with these gobies being a second intermediate host, as previously suggested by Cribb et al. [28], who reported that this is the most common life cycle form in the digenea, including Opisthorchioidea. Indeed, the life cycle of the only other species in the genus, *S. pearsoni,* includes a prosobranch snail that acts as a first intermediate host, harboring typical opisthorchioid cercariae, and this parasite also uses certain gobiiforme freshwater fishes (Eleotridae, also very diverse in the Solomon Islands) as secondary hosts [21]. The definitive hosts could be Terapontidae (*Mesopristes argenteus* (Cuvier, 1829), *M. cancellatus* (Cuvier, 1829), *Terapon jarbua* (Forsskal, 1775)) or a Kuhliidae (*Kuhlia marginata* (Cuvier, 1829), *Kuhlia rupestris* (Lacépède, 1802)), all of which prey on gobiiforme fishes [4]. In support of this idea, Cribb described that *S. pearsoni* cercariae respond to the presence of a fish fin only after direct contact with it and immediately adheres by the anterior end to posteriorly initiate penetration [21]. Conversely, the low occurrence of *S. cribbi* in specimens of *S*. *cynocephalus* caught in the Poro River during the wet season suggests that the observation of *S*. *cribbi* in this goby specimen might represent an infrequent event or an accidental infection of this host with infective cercariae. It would be interesting in future works to investigate Eleotridae of the Solomon Islands (such as *Hypseleotris cf guentheri* (Bleeker, 1875) since Cribb [21] has reported that Hypseleotris species are secondary intermediate hosts of *S. pearsoni*. Additional studies on the seasonal occurrence of *S. cribbi* will be fundamental to establishing its pattern of infection, considering that seasonal changes represent a combination of many factors that could influence the success of this parasitic relationship [22,29]. 

From a pathogenicity point of view, a number of parasitological surveys have reported digenetic trematodes causing tissue damage in freshwater fishes, thereby implicating them in the losses of fish by direct mortality or by increasing the susceptibility of infected fish to predation [30,31,32,33,34,35]. Regarding infections by *Stemmatostoma* spp. in the approximately ten fish species reported to be infected by these cryptogonimids, practically no histological alterations have been observed [11,21]. However, Cribb observed pathological alterations caused by *S. pearsoni* cercariae in the first intermediate host (snails) with destroyed reticulum, swollen areas of filament filled with plasma, and the epithelium drawn thin and sometimes broken [21]. In our study, histological analysis of the gill infected by trematodes was not performed. However, considering that the gills, in addition to being the major respiratory organ, play an important role in ionic balance and are the primary site of nitrogenous waste excretion [36], future studies addressing gill tissue damage will be necessary to determine the pathogenic aspect of this relationship.

In the taxonomic classification of cryptogonimid parasites, the 28S and ITS2 rDNA regions have proven to be reliable markers in studies addressing evolutionary aspects and species integrity [11,15,18]. The pairwise analysis of the multiple rDNA regions (28S, 5.8S, and ITS2) comparing the specimen obtained in the present study to previously available *S. cribbi* sequences revealed different levels of sequence variation among these rDNA sequences. Based on these studies, the few genetic differences observed between these two sets of sequences, with eight nucleotides over the 28S rDNA (3781 bp) and five nucleotides over the 5.8S and ITS2 (456 bp), led us to identify these specimens as *S. cribbi*. However, recent studies on Apocreadiidae and Bivesiculidae [19,37] showed that different species for which *COI* sequence data demonstrated substantial differences all had identical 28S sequences for corresponding specimens. They also showed that for these families, ITS2 and 28S are poor markers for closely related species. These characteristics have not been demonstrated for Cryptogonimidae, so further studies are needed on *COI* sequences to compare our specimens with other specimens previously identified as *Stemmatostoma* spp. Nevertheless, based on the results of our molecular analyses, we consider that our specimens are indeed *S. cribbi*, especially given the extensive morphological similarities between our specimens and adult *S. cribbi* [11].

The low genetic divergence observed between *S. cribbi* specimens collected from two widely-separated localities (Australia and Solomon Islands, 3280 km apart) indicates that geography has had little impact on genetic divergence. Our findings are consistent with previous studies in which samples of the same trematode species collected in distant geographic areas had very little or no genetic divergence in rDNA sequences [36,38,39]. Tropical island streams are considered fragmented habitats, isolated from one other (from the catchment area scale to the Pacific Ocean scale), and it may be surprising for *S. cribbi* to have such a wide geographic distribution. The high genetic similarity between the Ranongga Island and the Australian isolates of *S. cribbi* is likely a result of its highly specialized life cycle in different possible amphidromous hosts [4]. Indeed, freshwater fauna implicated in the parasite’s life cycle (snails and fish) are adapted to these distinctive habitats: the adults live, grow, and reproduce in the river, but the fish larvae spend 3 to 6 months at sea. This marine phase is highly important for the dispersal of individuals among different freshwater systems and for the colonization of new habitats. The parasite may be transported by the fish larvae during this marine dispersal phase. Fish larvae return to rivers after the marine phase. All the hosts of the parasite, including the definitive host (such as *Kuhlia marginata* or *Kuhlia rupestris*), the first intermediate hosts (snails such as *Neritina stumpffi* Boettger, 1890 or *Neritina canalis* Sowerby, 1825 [40]), and the secondary intermediate hosts (*Eleotris, Hypseleotris* or *Sicyopterus*—this paper) are present in the streams of the Solomon Islands. The cercariae may infect goby fish larvae external tissues once they emerge from the snail’s foot and may be carried over great distances during the marine dispersal phase of this amphidromous species. 

In conclusion, we report the first example of the *S. cribbi* parasite in a Solomon Islands goby, *S. cynocephalus*. Based on the literature, *S. cribbi* is a digenean trematode with a complex life cycle with at least two intermediate hosts, a snail as the first intermediate host and a Gobioid fish as the second intermediate host. Definitive hosts appear to be fish of the Terapontidae and the Kuhliidae families. All three families of hosts are present in the Solomon Islands, but it is the first time that a goby of the Sicydiinae subfamily was found as a secondary intermediate host. Indeed, up until now, gobies of the Eleotridae family have been considered to be the principal secondary intermediate host for *Stemmatostoma* parasites. Eleotridae are also present in the Solomon Islands; further studies are necessary to know whether the infection of *S. cynocephalus* by *S. cribbi* is accidental or commonly found in the Solomon Islands. Additional studies of the adult forms in the definitive hosts should contribute to a better understanding of the complete life cycle. Finally, the amphidromous life cycle of the secondary intermediate host could explain the wide geographic distribution of *S. cribbi*.

## Figures and Tables

**Table 1:** Cyst and encysted metacercaria measurements in µm. NA: not applicable; ventral suckers were not observed on 3 of the specimens.

Specimen Cyst		Encysted	Encysted	Circumoral	Oral Sucker	Ventral Sucker
	Diameter	Metacercaria Length	Metacercaria Width	Spine Length	Width	Width
1	113	165	48	30	16	15
2	131	231	55	29	23	NA
3	139	214	49	41	17	20
4	179	250	63	39	19	NA
5	115	209	46	28	20	NA
Mean	135.4	213.8	52.2	33.4	19	17.5
min	113	165	46	28	16	15
max	179	250	63	41	23	20

## Data Availability

The data set presented in this study is available upon reasonable request to the corresponding author.

## References

[1] Anggraini N., Karyadi B., Ekaputri R.Z., Zukmadini A.Y., Sastiawan R., Anggriani F. (2018). The population and habitat of mungkus fish (*Sicyopterus cynocephalus*) in Bengkenang Waters South of Bengkulu. J. Phys. Conf. Ser..

[2] Lord C., Bellec L., Dettaï A., Bonillo C., Keith P. (2019). Does your lip stick? Evolutionary aspects of the mouth morphology of the Indo-Pacific clinging goby of the *Sicyopterus* genus (Teleostei: Gobioidei: Sicydiinae) based on mitogenome phylogeny. J. Zool. Syst. Evol. Res..

[3] Keith P., Lord C., Maeda K. (2015). Indo-Pacific Sicydiinae Gobies: Biodiversity, Life Traits and Conservation.

[4] Keith P. (2003). Biology and ecology of amphidromous Gobiidae in the Indo-Pacific and the Caribbean regions. J. Fish Biol..

[5] Keith P., Lord C., Patzner R.A., Van Tassell J.L., Kovacic M., Kapoor B.G. (2011). Tropical freshwater gobies: Amphidromy as a life cycle. The Biology of Gobies.

[6] Lord C., Brun C., Hautecœur M., Keith P. (2010). Insights on endemism: Comparison of the marine larval duration estimated by otolith microstructural analysis of three amphidromous *Sicyopterus* species (Gobiidae: Sicydiinae) from Vanuatu and New Caledonia. Ecol. Freshw. Fish..

[7] Ebner B.C., Donaldson J.A., Allen G.R., Keith P. (2017). Visual census, photographic records and the trial of a video network provide first evidence of the elusive *Sicyopterus cynocephalus* in Australia. Cybium.

[8] Tigulu I.G., Ifuto’o M.R., Sheppard S. (2018). Ridges to Reef Conservation Plan Ghizo and Kolombangara Western Province, SOLOMON Islands.

[9] Keith P., Boseto D., Lord C. (2021). Freshwater Fish of the Solomon Islands.

[10] Mathews P.D., Bonillo C., Rabet N., Lord C., Causse R., Keith P., Audebert F. (2021). Phylogenetic analysis and characterization of a new parasitic cnidarian (Myxosporea: Myxobolidae) parasitizing skin of the giant mottled eel from the Solomon Islands. Infect. Genet. Evol..

[11] Miller T.L., Adlard R.D. (2020). *Stemmatostoma cribbi* n. sp. (Digenea: Cryptogonimidae) from Freshwater Fishes in the Wet Tropics Bioregion of Queensland, Australia. J. Parasitol..

[12] Miller T.L., Cribb T.H., Bray R.A., Gibson D.I., Jones A. (2008). Family Cryptogonimidae Ward, 1917. Keys to the Trematoda.

[13] Quintana M.G., De Núñez M.O. (2014). The life cycle of *Pseudosellacotyla lutzi* (Digenea: Cryptogonimidae), in *Aylacostoma chloroticum* (Prosobranchia: Thiaridae), and *Hoplias malabaricus* (Characiformes: Erythrinidae), in Argentina. J. Parasitol..

[14] Kmentová N., Bray R.A., Koblmüller S., Artois T., De Keyser E.L., Gelnar M., Vanhove M.P.M., Georgieva S. (2020). Uncharted digenean diversity in Lake Tanganyika: Cryptogonimids (Digenea: Cryptogonimidae) infecting endemic lates perches (Actinopterygii: Latidae). Parasit. Vectors.

[15] Miller T.L., Cribb T.H. (2007). Two new cryptogonimid genera *Beluesca* n. gen. and *Chelediadema* n. gen. (Digenea: Cryptogonimidae) from tropical Indo-West Pacific Haemulidae (Perciformes). Zootaxa.

[16] Miller T.L., Cribb T.H. (2013). Dramatic phenotypic plasticity within species of *Siphomutabilus* n. g. (Digenea: Cryptogonimidae) from Indo-Pacific caesionines (Perciformes: Lutjanidae). Syst. Parasitol..

[17] Martin S.B., Cutmore S.C. (2022). *Siphoderina hustoni* n. sp. (Platyhelminthes: Trematoda: Cryptogonimidae) from the Maori snapper *Lutjanus rivulatus* (Cuvier) on the Great Barrier Reef. Syst. Parasitol..

[18] Miller T.L., Cribb T.H. (2008). Eight new species of *Siphoderina* Manter, 1934 (Digenea, Cryptogonimidae) infecting Lutjanidae and Haemulidae (Perciformes) off Australia. Acta Parasitol..

[19] Cribb T.H., Bray R.A., Justine J.-L., Reimer J., Sasal P., Shirakashi S., Cutmore S.C. (2022). A world of taxonomic pain: Cryptic species, inexplicable host-specificity, and host-induced morphological variation among species of *Bivesicula* Yamaguti, 1934 (Trematoda: Bivesiculidae) from Indo-Pacific Holocentridae, Muraenidae and Serranidae. Parasitology.

[20] Kvach Y., Bryjová A., Sasal P., Winkler H.M. (2017). A revision of the genus *Aphalloides* (Digenea: Cryptogonimidae), parasites of European brackish water fishes. Parasitol. Res..

[21] Cribb T.H. (1986). The life cycle and morphology of *Stemmatostoma pearsoni*, gen. et sp. nov., with notes on the morphology of *Telogaster opisthorchis* Macfarlane (Digenea: Cryptogonimidae). Aust. J. Zool..

[22] Cuadros R.C., Rivadeneyra N.L.S., Malta J.C.O., Serrano-Martínez M.E., Mathews P.D. (2018). Morphology and surface ultrastructure of *Dadaytrema oxycephala* (Digenea: Cladorchiidae) with a new host record from Peruvian Amazon floodplain. Biologia.

[23] Ward R.D., Zemlak T.S., Innes B.H., Last P.R., Hebert P.D. (2005). DNA barcoding Australia’s fish species. Phil. Trans. R. Soc. B..

[24] Altschul S.F., Madden T.L., Schaffer A.A., Zhang J.H., Zhang Z., Miller W., Lipman D.J. (1997). Gapped BLAST and PSI-BLAST: A new generation of protein database search programs. Nucleic Acids Res..

[25] Hall T.A. (1999). BioEdit: A user-friendly biological sequence alignment editor and analysis program for Windows 95/98/NT. Nucl. Acids Symp. Ser..

[26] Tamura K., Stecher G., Peterson D., Filipski A., Kumar S. (2013). MEGA 6: Molecular evolutionary genetics analysis version 6.0. Mol. Biol. Evol..

[27] Ronquist F., Teslenko M., Van der Mark P., Ayres D.L., Darling A.A., Höhna S., Larget B., Liu L., Suchard M.A., Huelsenbeck J.P. (2012). MrBayes 3.2: Efficient Bayesian phylogenetic inference and model choice across a large model space. Syst. Biol..

[28] Cribb T.H., Bray R.A., Olson P.D., Littlewood D.T.J. (2003). Life cycle evolution in the digenea: A new perspective from phylogeny. Adv. Parasitol..

[29] Abdel-Gabe R., Abdel-Ghaffar F., Maher S., El-Mallah A., Al Quraishy S., Mehlhorn H. (2017). Morphological re-description and phylogenetic relationship of five myxosporean species of family Myxobolidae infecting the Nile tilapia *Oreochromis niloticus* (Perciformes: Cichlidae). Dis. Aquat. Org..

[30] Thatcher V.E., Varella A.B. (1980). Patologia de peixes da Amazônia brasileira. 2. Um tumor maligno das brânquias relacionado com as metacercárias de um trematódeo. Acta Amaz..

[31] Overstreet R.M., Curram S.S. (2004). Defeating diplostomoid dangers in USA catfish aquaculture. Folia Parasitol..

[32] Corrêa L.L., Souza G.T., Takemoto R.M., Ceccarelli P.S., Adriano E.A. (2014). Behavioral changes caused by *Austrodiplostomum* spp. in *Hoplias malabaricus* from the São Francisco River, Brazil. Parasitol. Res..

[33] Madrid R.R.M., Mertins O., Tavares-Dias M., Flores-Gonzales A.P., Patta A.C.M.F., Ramirez C.A.B., Rigoni V.L.S., Mathews P.D. (2021). High compliance and effective treatment of fish endoparasitic infections with oral drug delivery nanobioparticles: Safety of intestinal tissue and blood parameters. J. Fish Dis..

[34] Mathews P.D., Patta A.C.M.F., Gonçalves J.V., Gama G.S., Garcia I.T.S., Mertins O. (2018). Targeted drug delivery and treatment of endoparasites with biocompatible particles of pH-responsive structure. Biomacromolecules.

[35] Mathews P.D., Patta A.C.M.F., Madrid R.R.M., Ramirez C.A.B., Pimenta B.V., Mertins O. (2023). Efficient treatment of fish intestinal parasites applying membrane-penetrating oral drug delivery nanoparticle. ACS Biomater. Sci. Eng..

[36] Noga E.J. (2000). Fish Disease: Diagnosis and Treatment.

[37] Magro L., Cutmore S.C., Carrasson M., Cribb T.H. (2023). Integrated characterisation of nine species of the Schistorchiinae (Trematoda: Apocreadiidae) from Indo-Pacific fishes: Two new species, a new genus, and a resurrected but ‘cryptic’ genus. Syst. Parasitol..

[38] Lo C.M., Morgan J.A.T., Galzin R., Cribb T.H. (2001). Identical digeneans in coral reef fishes from French Polynesia and the Great Barrier Reef (Australia) demonstrated by morphology and molecules. Int. J. Parasitol..

[39] Nolan M.J., Cribb T.H. (2005). The use and implications of ribosomal DNA sequencing for the discrimination of digenean species. Adv. Parasitol..

[40] Abdou A., Lord C., Keith P., Galzin R. (2019). Phylogéographie de *Neritina stumpffi* Boettger, 1890 et *Neritina canalis* Sowerby, 1825 (Gastropoda, Cycloneritida, Neritidae). Zoosystema.

